# Infection Studies with Airway Organoids from *Carollia perspicillata* Indicate That the Respiratory Epithelium Is Not a Barrier for Interspecies Transmission of Influenza Viruses

**DOI:** 10.1128/spectrum.03098-22

**Published:** 2023-03-14

**Authors:** Ang Su, Miaomiao Yan, Suvarin Pavasutthipaisit, Kathrin D. Wicke, Guntram A. Grassl, Andreas Beineke, Felix Felmy, Sabine Schmidt, Karl-Heinz Esser, Paul Becher, Georg Herrler

**Affiliations:** a Department of Infectious Diseases, Institute of Virology, University of Veterinary Medicine Hannover, Foundation, Hannover, Germany; b Department of Pathology, University of Veterinary Medicine Hannover, Foundation, Hannover, Germany; c Department of Pathology, Faculty of Veterinary Medicine, Mahanakorn University of Technology, Bangkok, Thailand; d Institute of Zoology, University of Veterinary Medicine Hannover, Foundation, Hannover, Germany; e Institute of Medical Microbiology and Hospital Epidemiology, Hannover Medical School and German Center for Infection Research (DZIF), Hannover, Germany; f Center for Systems Neuroscience, Hannover, Germany; University of Prince Edward Island

**Keywords:** bat air-liquid interface cell culture, airway organoid, influenza virus, interspecies transmission

## Abstract

Bats are a natural reservoir for many viruses and are considered to play an important role in the interspecies transmission of viruses. To analyze the susceptibility of bat airway cells to infection by viruses of other mammalian species, we developed an airway organoid culture model derived from airways of Carollia perspicillata. Application of specific antibodies for fluorescent staining indicated that the cell composition of organoids resembled those of bat trachea and lungs as determined by immunohistochemistry. Infection studies indicated that *Carollia perspicillata* bat airway organoids (AOs) from the trachea or the lung are highly susceptible to infection by two different porcine influenza A viruses. The bat AOs were also used to develop an air-liquid interface (ALI) culture system of filter-grown epithelial cells. Infection of these cells showed the same characteristics, including lower virulence and enhanced replication and release of the H1N1/2006 virus compared to infection with H3N2/2007. These observations agreed with the results obtained by infection of porcine ALI cultures with these two virus strains. Interestingly, lectin staining indicated that bat airway cells only contain a small amount of alpha 2,6-linked sialic acid, the preferred receptor determinant for mammalian influenza A viruses. In contrast, large amounts of alpha 2,3-linked sialic acid, the preferred receptor determinant for avian influenza viruses, are present in bat airway epithelial cells. Therefore, bat airway cells may be susceptible not only to mammalian but also to avian influenza viruses. Our culture models, which can be extended to other parts of the airways and to other species, provide a promising tool to analyze virus infectivity and the transmission of viruses both from bats to other species and from other species to bats.

**IMPORTANCE** We developed an organoid culture system derived from the airways of the bat species *Carollia perspicillata*. Using this cell system, we showed that the airway epithelium of these bats is highly susceptible to infection by influenza viruses of other mammalian species and thus is not a barrier for interspecies transmission. These organoids provide an almost unlimited supply of airway epithelial cells that can be used to generate well-differentiated epithelial cells and perform infection studies. The establishment of the organoid model required only three animals, and can be extended to other epithelia (nose, intestine) as well as to other species (bat and other animal species). Therefore, organoids promise to be a valuable tool for future zoonosis research on the interspecies transmission of viruses (e.g., bat → intermediate host → human).

## INTRODUCTION

Bats are recognized as an important natural reservoir for a variety of zoonotic pathogens, including severe acute respiratory syndrome coronavirus (SARS-CoV)-1, SARS-CoV-2, Middle East respiratory syndrome coronavirus (MERS-CoV), Hendra virus, Ebola virus, and Marburg virus (1–6). The observation that bats develop no symptoms or only mild symptoms after infection with these viruses is largely unexplained (7). Influenza A viruses (IAVs) circulate in a broad range of avian and mammalian species and can cause disease in both humans and animals (8–10). Swine-origin influenza A (H1N1 subtype) viruses caused a pandemic in 2009 (11, 12) and avian-origin influenza viruses have been able to cross the species barrier several times (13, 14); thus, influenza viruses are a constant threat to the health of humans, poultry, and other animals (15, 16). Bats were found to harbor IAVs when genomic RNA of the subtypes H17N10 and H18N11 was identified by sequencing in the feces of a little yellow-shouldered fruit bat (Sturnira lilium) from Guatemala and a flat-faced fruit-eating bat (Artibeus planirostris) from Peru (17, 18). About 30% of fruit bats (Eidolon helvum) from Ghana were found to be serologically positive for avian IAV of the H9 subtype (19). In another study, 105 out of 1,202 oral samples from Egypt fruit bats tested positive for influenza A virus (20). These findings raised the question of bats being reservoir hosts for multiple influenza A viruses. The virus strain A/bat/Egypt/381OP/2017 (H9N2) was isolated in Egypt bats but is phylogenetically distinct from all other influenza A viruses. However, hemagglutinin (HA) and neuraminidase (NA) gene analyses revealed that this particular virus was closely related to avian H9N2 viruses. Interestingly, this finding agrees with a recent report that Egyptian fruit bats are most likely not susceptible to the avian H9N2 subtype but can be infected with bat-derived H9N2 (21). These findings raised questions about an evolutionary relationship between avian and bat-borne influenza A viruses (22). However, the adaptation process of influenza viruses in the bat respiratory tract is largely a mystery. The species Carollia perspicillata is one of the most common fruit bat species in New World bats (23, 24). Animals of this species are a natural reservoir for influenza viruses of the subtype H18N11 (18, 25). In addition, lung epithelial cells of the C. perspicillata species were shown to be permissive for avian and human influenza viruses (26). Therefore, C. perspicillata provides a good model to analyze the infection and transmission of influenza viruses from bats to other species and from other species to bats, as well as to study infection by bat influenza viruses (25).

The respiratory epithelium acts as a physical, chemical, and immunological barrier to prevent the invasion of microorganisms (27, 28). IAVs overcome the epithelial barrier by infecting the ciliated cells (29). To initiate the replication cycle, influenza viruses make use of the surface glycoprotein hemagglutinin, which has sialic acid-binding activity. Human and swine influenza viruses preferentially recognize sialic acid linked to Gal via an alpha 2,6 linkage; avian-origin influenza viruses prefer alpha 2,3-linked sialic acids (30, 31).

Currently, only a limited number of research models are available to analyze the susceptibility of bat airway cells to infection by viruses and other pathogens. Experimental oronasal infection of Seba’s short-tailed bats (C. perspicillata) with bat influenza A virus H18N11 has been reported (25). Experimental bats are not readily available on a routine basis. Although immortalized bat cell lines are a useful *in vitro* tool to analyze bat immunology and virus-host interactions, most of these cell lines poorly recapitulate the histology of the bat airway epithelium (26, 32).

Recent advances in stem cell biology have enabled the *in vitro* growth of 3D organoids that recapitulate the essential compartments of their counterpart organs *in vivo* (33, 34). A tiny piece of an organ biopsy specimen can grow into a self-organizing 3D organoid under optimized conditions (35, 36). Another alternative system is an air-liquid interface (ALI) culture. In this system, airway epithelial cells are collected and cultivated on collagen-coated filter supports until they are differentiated. The presence of multiple cell types (mucus-producing, ciliated, basal, and club cells) that are characteristic to the respiratory tract makes this system a good tool for isolating other respiratory pathogens and investigating interactions between hosts and pathogens. Selective access to both the apical and basolateral domain of ALI cultures allows study of the polarity (apical/basolateral) of virus entry and release. Due to the small size of bats, it is impossible to collect and isolate enough primary epithelial cells from a single animal to cultivate an ALI culture. By using organoid cultures, bat stem cells derived from airway organoids can be expanded and grown into bat trachea and lung ALI cultures.

Considering the recent findings of influenza viruses and specific antibodies in multiple bat species, bats may be significantly involved in influenza A virus transmission cycles and may be potential vessels for reassortment processes. To investigate the infection and transmission of influenza viruses in the bat airway system, we first explored two swine IAV strains, A/sw/Bad Griesbach/IDT5604/2006 (H1N1) and A/sw/Herford/IDT5932/2007 (H3N2). These viruses were chosen (rather than avian viruses) because we previously characterized them for their replication properties in porcine ALI cultures (avian ALI cultures are not yet available) (37, 38). By infecting bat ALI cultures with porcine viruses, it became possible to determine and compare the efficiency of infection in the swine and bat cell system. Here, we have developed an airway organoid culture model derived from the trachea and lung of the New World bat species C. perspicillata. We used the organoids and an air-liquid interface culture system derived from them for infection by two porcine influenza viruses. These well-differentiated airway epithelial cells were highly susceptible to infection even though they contained only a small amount of alpha 2,6-linked sialic acids, the preferred receptor determinant for mammalian influenza viruses. In contrast, these bat cells were rich in alpha 2,3-linked sialic acids, the receptor determinant for avian influenza viruses. Our culture models are promising tools for future zoonosis research on bat-related virus transmission.

## RESULTS

### Characterization of the *C. perspicillata* airway system.

**FIG 1:**
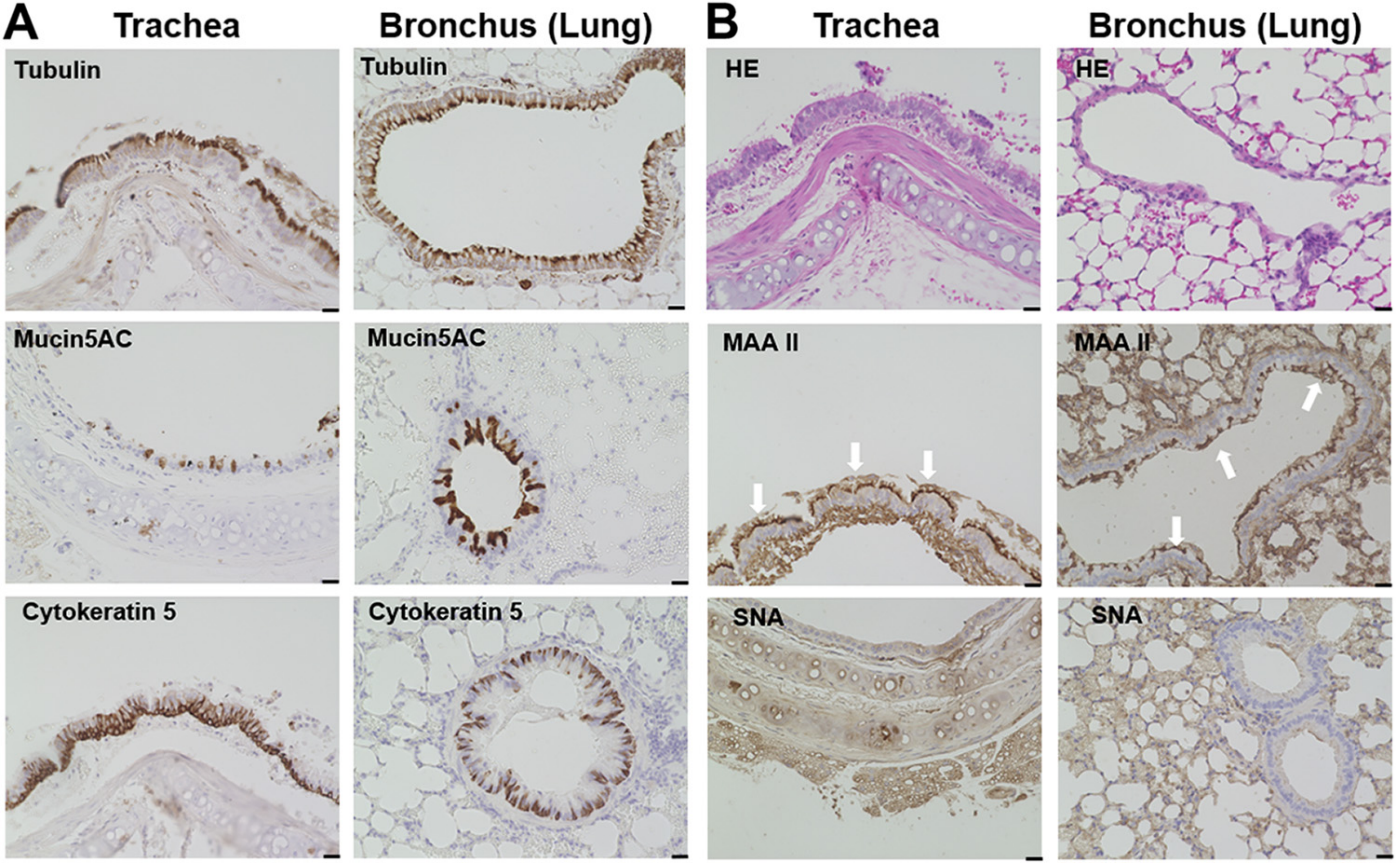
Characterization of the Carollia perspicillata airway system. Trachea and bronchus (lung) tissue were prepared from *C. perspicillata* bats and used for histological and immunohistochemistry staining. (A) Cells were visualized with antibodies against acetylated tubulin (ciliated cells, upper panels), mucin 5AC (mucin-producing cells, middle panels), or cytokeratin 5 (basal cells, lower panels). (B) Samples were subjected to hematoxylin and eosin (H&E) staining (upper panels) or lectin staining (middle and lower panels). Maackia amurensis agglutinin II (MAA II) was used to detect alpha 2,3-linked sialic acid (middle panels), and Sambucus nigra agglutinin (SNA) was used to detect alpha 2,6-linked sialic acids (lower panels). Scale bar = 20 μm. Photographs and images are representative of at least five similar images.

**FIG 2:**
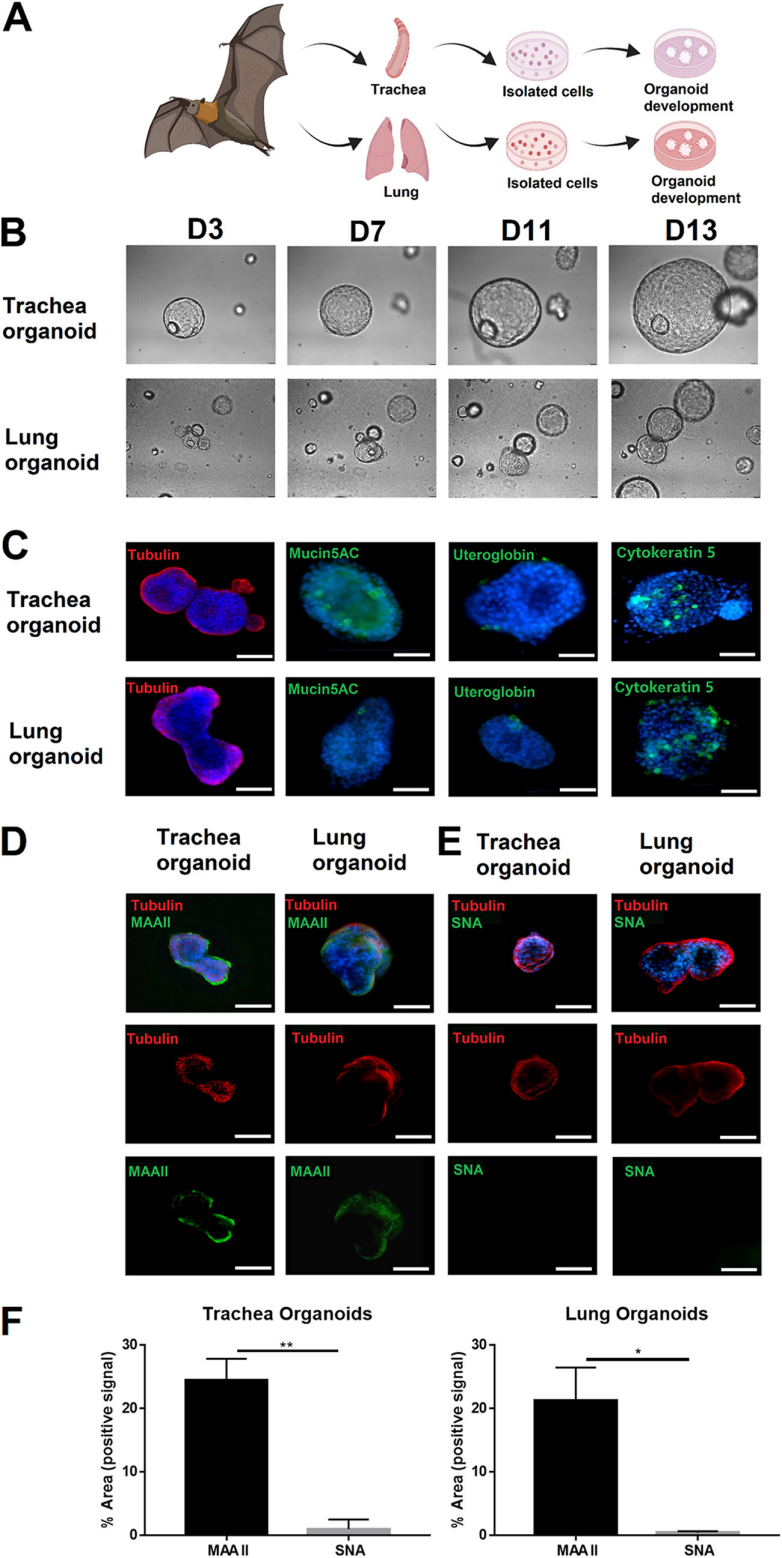
Characterization of Carollia perspicillata bat airway organoids (AOs). Several lines of bat airway organoids were generated and characterized. (A) Schematic of the generation of bat trachea and lung organoids. (B) Bright-field photographs of lung and trachea organoids at different times during development (3, 7, 11, 13 days). (C) After 7 days, the organoids were analyzed by immunofluorescence microscopy for the presence of cells which are characteristic of the respiratory epithelium (from left to right): ciliated (tubulin staining, red), mucus-producing (MUC5AC, green), club cells (uteroglobin, green), and basal cells (cytokeratin 5). (D and E) Co-staining of ciliated cells (red) and sialic acid-expressing cells (green). (D) Alpha 2,3-linked sialic acids were detected by MAA II staining and (E) alpha 2,6-linked sialic acids by SNA staining. (F) Distribution of alpha 2,3-linked and alpha 2,6-linked sialic acid in bat airway organoids was quantified by pixel counting using ImageJ software. Bars represent mean values from five pictures per well. Error bars indicate standard deviations. Scale bar = 50 μm. Photographs and images are representative of at least five similar images.

Molecular characterization of host-pathogen interactions depends on appropriate tissue culture systems. Therefore, we established a culture system of bat airway epithelial cells derived from the species C. perspicillata. To evaluate whether the cultures matched with the characteristic features of the airway epithelium of the respective host, we first analyzed trachea and lung tissue sections from C. perspicillata by histological methods and immunofluorescence microscopy. In both the trachea and bronchus (lung), ciliated cells were the predominant cell type (Fig. 1A, upper panels). Mucus-producing cells were found both in the trachea and lower respiratory tract (Fig. 1A, middle panels). Basal cells, which play an important role during differentiation, were detected using the cytokeratin 5 marker and were found mainly in the subepithelial compartment (Fig. 1A, lower panels). Histological staining indicated that the bat airway epithelium shows a characteristic appearance of pseudostratified ciliated columnar epithelium (Fig. 2B, upper panels). Because sialic acids are a receptor determinant for influenza viruses, the sialic acid distribution was determined by lectin staining. Staining with Maackia amurensis agglutinin II (MAA II) revealed that alpha 2,3-linked sialic acid was present throughout the whole trachea and lower respiratory tract (Fig. 1B, middle panels). It was mainly localized at the apical surface of the airway epithelium. In contrast, after staining with Sambucus nigra agglutinin (SNA), fluorescent signals were hardly detectable, indicating that the epithelial cells contain only small amounts of alpha 2,6-linked sialic acid (Fig. 1B, lower panels).

### Generation and characterization of *C. perspicillata* bat airway organoids.

Based on the organoid generation experience (33, 39) and recent developments in human airway organoids (34, 40), we established several lines of bat airway organoids. Briefly, small pieces of bat trachea and lung tissue were collected to isolate bat epithelial cells. Isolated cells were embedded in basement membrane matrix (Fig. 2A). Under optimized conditions, trachea 3D organoids and lung 3D organoids formed within 13 days (Fig. 2B). After a differentiation period of 7 days, AOs showed the characteristic appearance of a pseudostratified airway epithelium containing four main cell types (Fig. 2C): ciliated cells identified by staining of tubulin, mucus-producing cells detected by antibodies specific for mucin5AC, and club and basal cells visualized via the marker proteins uteroglobin and cytokeratin 5, respectively, by immunofluorescence microscopy (Fig. 2C). Organoids are self-organizing in three-dimensional culture due to their self-renewal and differentiation capacities; the presence of Ki67-positive cells is consistent with this property (data not shown). The apical side of most ciliated cells in bat AOs faces the lumen of the spheroid structures (Fig. 2D and E). The bat AOs were also analyzed for the distribution of sialic acids. Similar to the findings with natural airway tissue samples (Fig. 1), AOs were readily stained by MAA II but hardly stained by SNA. Co-localization of MAA II signals and ciliated cells indicated abundant expression of alpha 2,3-linked sialic acids on the surface of this cell type (Fig. 2D). A quantification analysis of sialic acid expression (Fig. 2F) revealed comparable expression patterns of alpha 2,3-linked and alpha 2,6-linked sialic acids on C. perspicillata airway organoids. These data show that the bat trachea and lung organoid systems have been successfully developed. Our results show that the AOs have the characteristics expected for a differentiated bat airway epithelium.

### Bat trachea and lung organoids support swine influenza virus replication.

**FIG 3:**
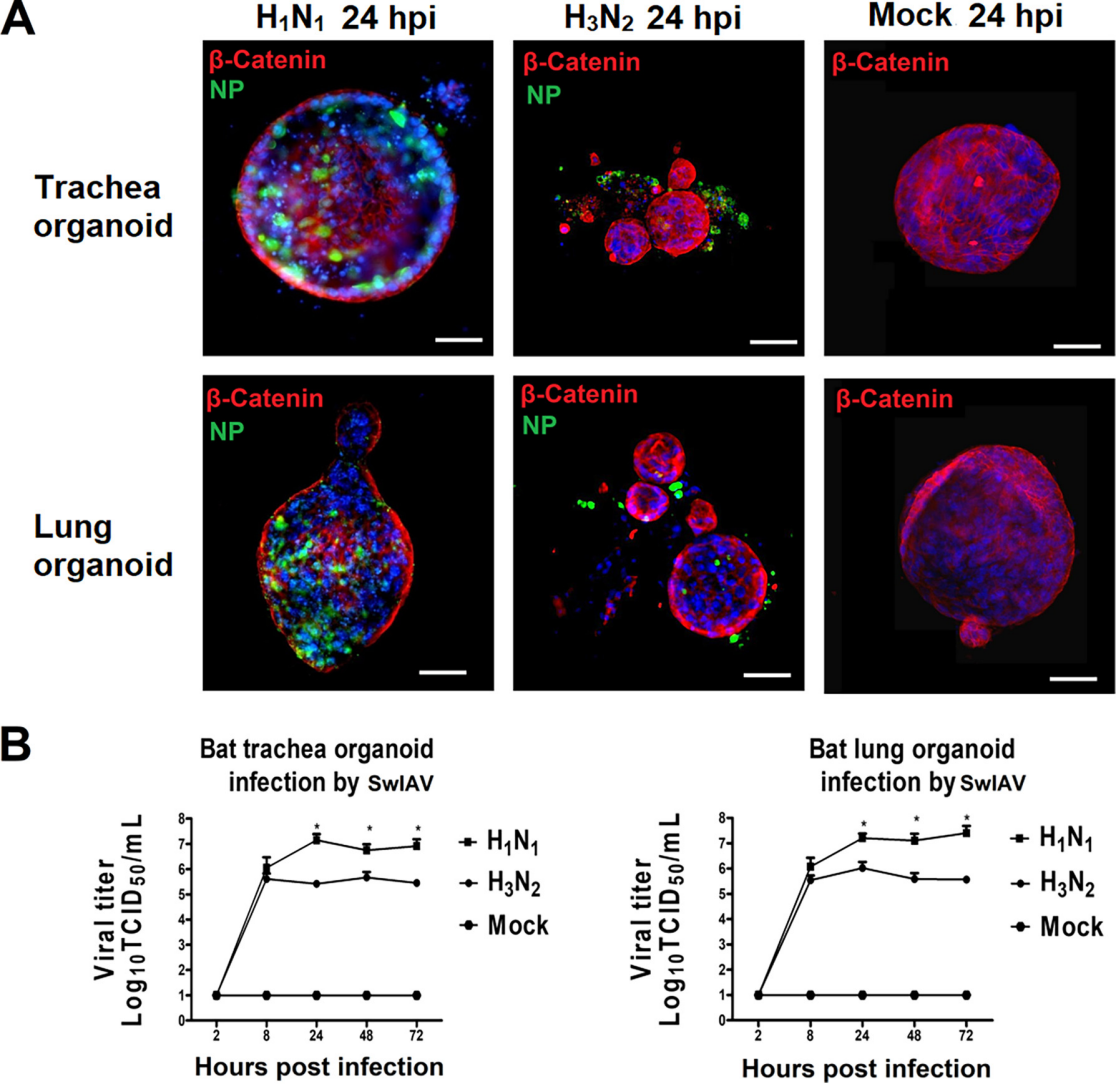
Infection of *Carollia perspicillata* bat airway organoids by swine influenza viruses (SwIAV). Bat AOs were infected by two swIAVs of the H1N1 or H3N2 subtypes, respectively, as described in Materials and Methods. (A) At 24 h postinfection (hpi), infected cells were visualized by immunostaining of the nucleoprotein (NP) (green); tight junctions were stained by an antibody against β-catenin (red), and nuclei were stained by DAPI (4′,6-diamidino-2-phenylindole) (blue). (B) To investigate the replication kinetics of the two swIAVs in bat AOs, samples were taken at different times postinfection, and the generation of infectious virus was determined by an infectivity assay. TCID_50_, 50% tissue culture infective dose. Scale bars = 50 μm. Values are shown as mean ± standard error of the mean (SEM). All experiments were performed at least three times. Some of the error bars were too small to be printed. Photographs and images are representative of at least five similar images. Significance was analyzed with Tukey’s multiple-comparisons test using GraphPad Prism 8 software (*, *P* < 0.05).

To gain knowledge whether bat AOs can support IAV infection, we analyzed infection by two swine influenza viruses (swIAVs) belonging to the H3N2 and H1N1 subtypes. The H3N2 virus is a virulent strain, whereas H1N1 has been characterized in animal experiments as a low-virulence virus (38). In our infection study, the AOs were collected after 4 days of differentiation and infected by the two swine influenza virus strains. As shown in Fig. 3A, viral antigen was detectable by immunofluorescence microscopy at 24 h postinfection (hpi), indicating that both trachea organoids and lung organoids were highly susceptible to the two swine IAVs. Higher levels of viral antigen (nucleoprotein, NP) were observed in the trachea and lung organoids infected by the H1N1 virus compared to those infected with H3N2 (Fig. 3A, left panels). The H3N2 virus induced a more detrimental effect on organoids, resulting in much smaller spheres compared to mock-infected AOs and AOs infected by H1N1 (Fig. 3A, middle panels). To gain information about the efficiency of infection, we determined the virus replication kinetics (Fig. 3B). The amplification of infectious virus over a period of 3 days had a similar time course for both viruses in trachea and lung organoids. However, the amount of swIAV generated by H1N1 was significantly higher than that generated by swIAV H3N2 (Fig. 3B). At 24 to 72 hpi, the differences were >10-fold and highly significant (*P* < 0.01; Fig. 3B).

### An air-liquid interface cell culture system for differentiated bat trachea and lung epithelial cells.

**FIG 4:**
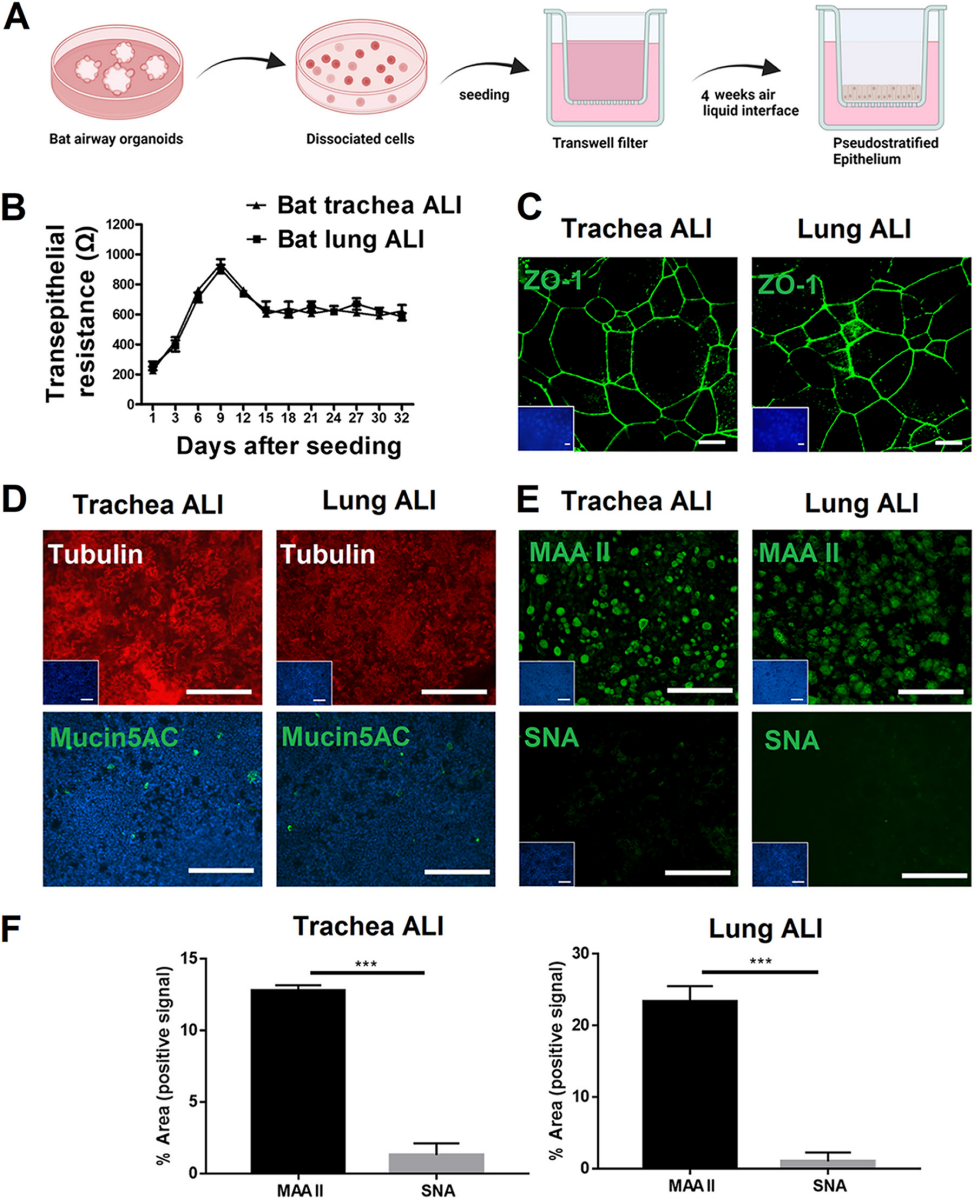
Generation of bat trachea and bat lung air-liquid interface (ALI) cell cultures. To gain more insights into bat airway systems, we generated bat trachea and lung air-liquid interface cell cultures. (A) Schematic of the generation of bat ALI cultures derived from AOs. In brief, bat organoids were dissociated into single cells. The cells were seeded onto Transwell filters until they reached confluence, followed by the removal of the apical medium, which induces the differentiation process over a time period of up to 4 weeks. (B) The barrier function of the polarized epithelial cells was assessed by measuring the transepithelial electrical resistance (TEER). (C) Tight junctions were stained by an antibody against ZO-1. (D) Ciliated cells were immunostained with tubulin (red); mucus-producing cells were immunostained using the mucin 5AC antibody (green). (E) Sialic acids were visualized by lectin-staining (green): MAA II interacts with alpha 2,3-linked sialic acids, and SNA with alpha 2,6-linked sialic acids. Nuclei were stained with DAPI (blue), Scale bar = 50 μm. (F) Expression of alpha 2,3-linked and alpha 2,6-linked sialic acid on bat trachea and lung ALI were quantified by pixel counting using ImageJ software. Bars represent mean values from five pictures per well.

As has been reported for intestinal organoids (41, 42), most airway organoids have an inside-out orientation (34, 43), i.e., the apical side of the epithelium faces the lumen of the spherical organoid structures. This characteristic makes it difficult to infect epithelial cells via the apical surface. To overcome this problem, we used epithelial cells derived from airway organoids to generate filter-grown air-liquid interface cultures. Bat airway 3D organoids were enzymatically dissociated into single cells, seeded on 0.4-μm Transwell filters, and cultured in AO growth medium. When the cells had become confluent, they were incubated with differentiation media under air-liquid interface conditions. After about 4 weeks, the differentiation process was completed (Fig. 4A). To determine whether trachea and lung ALIs can maintain the barrier function, we determined the transepithelial electrical resistance (TEER). On day 9 after cell seeding, the TEER values reached their peak and remained stable until the end of the observation period (Fig. 4B). The barrier function was also demonstrated by positive staining for the tight junction marker ZO-1 (Fig. 4C) on trachea and lung ALIs on day 32. These results show that bat ALI cultures maintain the integrity of the epithelial structure, including the barrier function. As shown in Fig. 4D, after an incubation period of about 4 weeks, the ALI cultures were well-differentiated as indicated by the large number of ciliated cells. Immunofluorescent staining also revealed the presence of mucus-producing cells (Fig. 4D). These results demonstrate that the established bat trachea and lung ALI cultures have the characteristic features of a well-differentiated airway epithelium.

We also investigated the expression of sialic acids on the epithelial cells of bat trachea ALI and lung ALI cultures by lectin staining. The distribution of these potential influenza virus receptors on the bat ALI cultures was similar to that of bat AOs (Fig. 4E, F), i.e., sialic acids were mainly present in the alpha 2,3-linkage type.

### Bat ALI cultures are susceptible to infection by swIAV.

**FIG 5:**
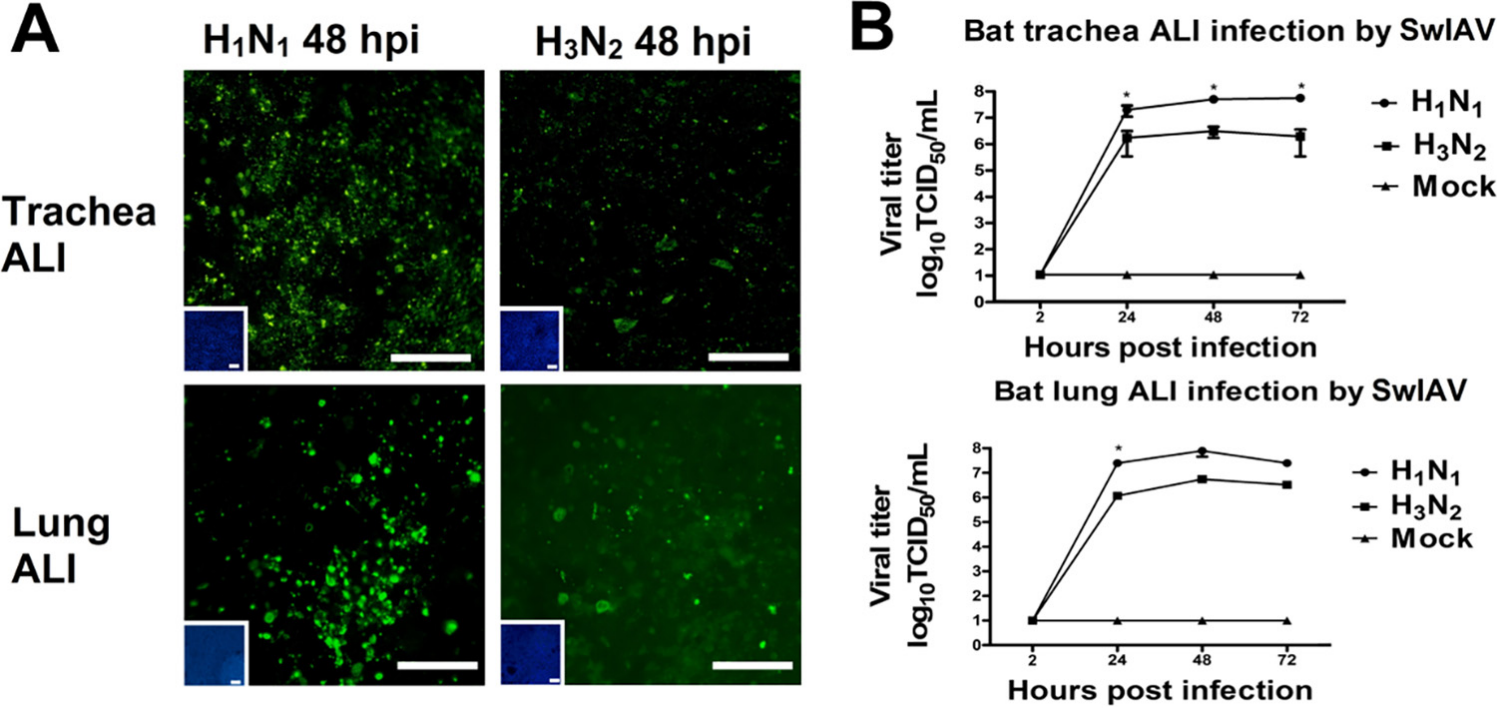
Infection of bat ALI cultures by swine influenza viruses. Bat ALI cultures were infected by two swIAVs of the H1N1 or H3N2 subtypes, respectively, as described in Materials and Methods. (A) Infected cells were visualized by immunostaining of the influenza virus NP (green). Scale bars = 50 μm. (B) For the replication kinetics of the two swIAV strains in bat ALI, samples were taken at different time points postinfection and the generation of infectious virus was determined by virus titration. Values are shown as mean ± SEM. All experiments were performed at least three times. Some of the error bars were too small to be printed. Significance was analyzed with Tukey’s multiple-comparisons test using GraphPad Prism 8 software (*, *P* < 0.05).

As described above for bat AOs, we also analyzed bat airway ALI cultures for their susceptibility to infection by swine influenza viruses. Trachea and lung ALI cultures grown on Transwell filters were inoculated via the apical filter chamber by swIAV H1N1 or swIAV H3N2 virus, respectively. Over an infection period of 72 h, the course of infection was monitored at daily intervals by fluorescence microscopy to visualize infected cells and by titration of the amount of infectious virus released into the apical supernatant. Both trachea and lung ALI cultures were readily infected by the two swIAVs. However, as in the infections of bat organoids (Fig. 3), the number of cells containing virus antigen was higher in the case of the H1N1-infected ALI cells (Fig. 5A). Also, more infectious virus was released from H1N1-infected cells. The difference between the two viruses was more than 10-fold and was statistically significant (Fig. 5B).

## DISCUSSION

Compared to mammalian and avian IAVs of the subtypes H1 to H16, little is known about the bat viruses of the subtypes H17 and H18. This lack of knowledge is not surprising considering the fact that no IAVs of these subtypes have been isolated from bats. The viruses known so far have been identified by sequencing genomic RNA from fecal samples (18, 44). This information has been used to generate virus by reverse genetics (45, 46). The lack of virus isolates may be partially due to the lack of appropriate cell systems. More recently, only one virus (A/bat/Egypt/381OP/2017) has been isolated from an Egyptian fruit bat (Rousettus aegyptiacus) which is distantly related to avian H9N2 viruses (20, 21). While this virus may have been transmitted from an avian species to bats, the origin of the H17N10 and H18N11 viruses is unknown. It is not known whether they originated from the transmission of a virus from other species or whether bat viruses spread to other species at some time point (20, 22).

For a better understanding of the biology of bat IAVs, it is necessary to obtain more virus isolates. For this purpose and for the characterization of isolates, appropriate cell culture models are necessary. The generation of airway organoids is an important step in this direction. AOs display the characteristic features of a differentiated airway epithelium (34, 40, 43). This tool will enable us to obtain reliable information about the replication of bat influenza viruses. It will also allow us to analyze the interspecies transmission.

We used AOs to analyze the ability of swine IAVs to replicate in bat airway epithelial cells. Our results demonstrate that the airway epithelium is highly susceptible to infection by swine IAV. These two swine virus strains were chosen because they have previously been analyzed for their replication in porcine precision-cut lung slides and porcine ALI cultures (37, 38). It turned out that these viruses amplified in bat ALI cultures as efficiently as in their porcine counterpart. In contrast to the H1N1 virus, H3N2 induces a detrimental effect, which may explain why a smaller amount of virus was released from this strain. The molecular reasons behind this difference are not known but, interestingly, they also appear to be valid in the *Carollia* cell system.

Thus, the airway epithelium is not a species barrier for transmission of swine IAV. This finding may be surprising because bat airway cells contain mainly alpha 2,3-linked sialic acid and only small amounts of the alpha 2,6-linkage type, the preferred binding partner of the hemagglutinin of swine influenza viruses. However, resialylation experiments show that influenza viruses only require very small amounts of sialic acid for successful initiation of infection (47). Furthermore, even sialic acids of an unfavorable linkage type can be used for infection if they are present in a sufficient amount on the cell surface, as is the case with the alpha 2,3-linked sialic acid in bat airway cells. The efficient binding of influenza viruses to sialic acids is due to the presence of a large number of hemagglutinin molecules on the surface of each virion, which allows a multivalent interaction with sialoglyco-conjugates. In a recent report, it was shown that the multivalent interactions enable influenza viruses to also involve low-affinity sialic acid receptors if the density of high-affinity receptors is low, and thus to enhance their binding efficiency and infection of cells (48). Because of the abundance of alpha 2,3-linked sialic acid, the preferred binding partner of the HA protein of avian IAV, it is reasonable to assume that bat airway epithelial cells are also susceptible to infection by avian viruses and thus are not a species barrier for these viruses. This assumption is supported by a recent study demonstrating that the lung epithelial cells of three different bat species (E. helvum, C. perspicillata, and Tadarida brasiliensis) express large amounts of alpha 2,3-linked sialic acid receptors and were more permissive to avian than human influenza viruses (26). In the latter study, similar amounts of alpha 2,3-linked and alpha 2,6-linked sialic acid receptors were detected in the lung epithelial cells of C. perspicillata and E. helvum, while expression of alpha 2,6-linked sialic acid receptor was barely detectable in T. brasiliensis. These results suggest that different bat species can significantly differ in the expression of different sialic acid receptors. The apparent difference in expression of alpha 2,6-linked sialic acid receptor in the previously studied lung epithelial cell line and the trachea and lung organoid cell cultures (this study) of *C. perspicillata* may be due to differences in origin, complex composition, complicated sorting mechanisms during polarized stages, and differentiation stages of the investigated epithelial cells (49, 50).

The susceptibility of the bat airway epithelium to infection by IAVs of other species does not necessarily imply that bats of the species C. perspicillata are susceptible to infection by these viruses. The H9N2-related virus that was isolated from R. aegyptiacus has been reported to infect bats of this species (21), whereas avian H9N2 viruses were unable to initiate a productive infection. Unlike the bats in South America or Egypt, 26 bat species from Central Europe were shown to be seronegative for influenza or influenza-like viruses, indicating that not every bat species can be expected to behave in the same way with respect to infection susceptibility (51). The different expression levels of viral proteins (PB1, M1, and NP) in three different bat cell types (C. perspicillata, E. helvum, and T. brasiliensis) separately infected with human influenza virus strains also demonstrates that different bat species may differ in their susceptibility to infection (26). It cannot be predicted whether bats of the species C. perspicillata are susceptible to infection by swine IAV. However, if they are resistant to infection, it is not because the respiratory epithelial cells are resistant to infection; the cause would have to be sought in another stage of the infection, e.g., innate immunity. For instance, bats apply a unique antiviral response by inducing a strong interferon response and controlling pro-inflammatory cytokine expression, which can limit MERS-CoV and Marburg virus infection (52, 53). Apart from sialic acids as receptor determinants, the influence of additional factors, including cellular proteases such as TMPRSS2 (transmembrane protease, serine 2) and TMPRSS4 (transmembrane protease, serine 4), on activating influenza viruses can be hypothesized (54, 55). Different expression patterns of TMPRSS2 and TMPRSS4 in bat airway organoids might affect the infection of different bat species (56).

The advantage of the AO system is that it provides an almost unlimited supply of airway epithelial cells that can be used for differentiation and infection experiments. Because the apical surface of the epithelial cells commonly faces the lumen of the AO spheres, the apical plasma membrane, a frequent primary target site for pathogens, is not readily accessible for infection experiments. This problem can be overcome by using the AOs to generate filter-grown air-liquid interface cultures. In the future, it will be interesting to extend this tool and generate AOs not only from the tracheal or bronchial epithelium but also from other parts of the respiratory tract, e.g., the nasal epithelium and the intestinal tract. In this way, it should be possible to obtain a comprehensive picture of the infection of the respiratory tract and intestine of C. perspicillata by IAV. It will be especially interesting to apply these culture models for isolating and characterizing bat IAVs.

Experiments with traditional ALI cultures are limited to animal species from which airway samples are readily available, e.g., animals from slaughterhouses. By applying this culture method to AOs, infection of differentiated airway epithelial cells is now also feasible for species which are less accessible for infection. We chose *Carollia perspicillata* to set up this infection model because a colony of this species is available at the University of Veterinary Medicine Hannover. This species is a natural reservoir for the H18N11 subtype of bat influenza viruses (18, 25). Therefore, AOs of *C. perspicillata* are a promising tool to analyze the replication of bat influenza viruses in the future.

For our study, we used the airways of three animals which were euthanized for experimental analysis of bat brains. This shows that a very small number of animals is sufficient to successfully establish an AO system. Because it uses freshly dead animals, the method is even applicable for animals that are not used for experimental research. Overall, AOs promise to become a tool that will allow us to perform infection experiments with pathogens that have been not previously feasible. In this way, AOs can become available not only from C. perspicillata but also from many different bat species. In the same way, AOs can be generated from species which serve as intermediate hosts in the transmission of viruses from bats to humans. This is an intriguing aspect for future zoonosis research.

## MATERIALS AND METHODS

### Establishment of *Carollia perspicillata* bat airway organoids.

Tissue extractions of mature *C. perspicillata* from an open colony at the Institute for Zoology were approved by the university’s local authorities under the license number TiHo-T-2019-7 and were in accord with German laws for animal protection. *C. perspicillata* species were kept and bred under biosafety level 2 conditions. These bats were free of rabies virus and ectoparasites. Weaned animals of both sexes were deeply anesthetized with isoflurane and subsequently decapitated. Fresh bat trachea and lung tissue samples were collected. Experimental bats were tested to be free of rabies virus. The generation of *C. perspicillata* bat airway organoids was based on a protocol reported for human cells (39, 40). In brief, a small piece of bat trachea or lung, respectively, was washed three times in cold phosphate-buffered saline (PBS). Then, the tissue was chopped and digested with 5 mg/mL protease in incubation medium including Dulbecco’s modified Eagle’s medium (DMEM)/F12 (Gibco) and antibiotics overnight. On the second day, 5% fetal bovine serum was added to the digestive suspension to terminate the digestion and the sample was passed through a 100-μm strainer. After centrifugation at 300 × *g*, single trachea and lung cells were collected and embedded in 60% basement membrane Matrigel (Corning). After solidification, 400 μL organoid growing medium including a 1:1 mixture of L-WNR condition medium and additives EGF (50 ng/mL, Peprotech, Inc.), A83-01 (500 nM, Peprotech, Inc.), SB 202190 (10 μM, Peprotech, Inc.), and B27 supplement (Gibco) was added, and organoids were cultured at 37°C in a humidified 5% CO_2_ atmosphere for 2 weeks. The organoids were passaged every 2 weeks at a ratio of 1:4. Bright-field images of organoids were captured using a Nikon Eclipse Ti-S microscope (Nikon).

### Establishment of a culture of well-differentiated bat airway epithelial cells.

This protocol is based on previous publications (34, 37, 40, 57). In brief, organoids were collected and dissociated into single cells by TrypLE in a 37°C water bath for 5 min. Approximately 2.5x10^5^ cells were seeded on a collagen-coated Transwell filter in organoid growth medium for the first 3 days. After 3 days cultivation, cells were washed with warm Hanks’ Balanced Salt Solution (HBSS) and changed to bat ALI medium in the apical (200 μL) and basolateral (600 μL) compartment consisting of DMEM/F12 (Gibco) and BEGM (Lonza) at a 1:1 ratio with additives as described previously (58, 59). When the cells had become confluent, the Transwell filters were maintained under air-liquid interface conditions for 4 weeks in bat ALI medium at 37°C in a humidified 5% CO_2_ atmosphere. During the 4 weeks differentiation period, the medium was changed every 3 days and the filters were washed once per week by HBSS. To assess the integrity of bat airway ALI cells, the TEER was determined every 3 days using a Millicell ERS-2 Voltohmmeter (Millipore) according to the manufacturer’s instructions.

### Cells and viruses.

Two strains of swine influenza viruses were used in this study: strain A/sw/Bad Griebach/IDT5604/2006 is a low-virulent virus of subtype H1N1, and strain A/sw/Herford/IDT5932/2007 belongs to subtype H3N2 and has been shown in animal experiments to be virulent for pigs (37, 38). Virus propagation and titration was performed in Madin-Darby canine kidney cells (MDCK, CRL-2936, American Type Culture Collection [ATCC]). Cells were grown in DMEM supplemented with 10% fetal calf serum.

### Virus infection of *Carollia perspicillata* bat airway organoids.

Prior to infection, the medium of the bat AOs was changed from growth medium to differentiation medium for at least 4 days. On the day of infection, bat fetal and lung organoids were sheared gently and collected in 50-mL Falcon tubes (Greiner) on ice for at least 1 h. Then, the Matrigel was removed by centrifugation at 300 × *g*. The organoid pellets were separated into three equal volumes by weight. Subsequently, the cells of two of these volumes were infected by either of the two swIAV variants separately at an MOI of 1 for 1 h at 37°C. The cells of the third volume were mock-infected by medium for 1 h in parallel. After an adsorption time of 1 h, the infected and mock-infected organoids were washed three times. Washed organoids were re-embedded in Matrigel with DMEM/F12 basic medium. At the indicated harvest time point, the samples in Matrigel were suspended in 150 μL cold PBS. Finally, 150 μL resuspended medium of each sample was collected after centrifugation and subjected to a viral titration assay. Infected and mock-infected samples were collected for immunofluorescent staining.

### Virus infection of bat airway ALI cultures.

Prior to infection, the well-differentiated bat trachea epithelial cells (bat trachea ALI) and lung epithelial cells (bat lung ALI) were washed three times with warm PBS. The filter-grown cells (approximately 5 × 10^5^ cells per well) were infected with diluted virus inoculum from the apical compartment at an MOI of 1 for 1 h at 37°C. Control cells were mock-infected with PBS. After unbound virus had been removed by washing, the infected and mock-infected filters were maintained with 600 μL bat ALI medium under air-liquid interface conditions for 3 days at 37°C and 5% CO_2_. Every 24 h, volumes of 100 μL medium were applied to the apical chamber, shaken for 30 min, and collected for endpoint titration to quantify the amount of produced infectious virus as previously described (37, 60).

### Histological and immunohistochemical examination.

For characterization of the bat airway, fresh bat trachea and lung from the University of Veterinary Medicine Hannover, Institute of Zoology as previously indicated were obtained separately and fixed in 10% neutral buffered formalin. As described previously (61, 62), formalin-fixed paraffin-embedded samples were sectioned at 2-μm thickness and prepared for hematoxylin and eosin (H&E) staining and immunohistochemistry (IHC) analysis using a standard protocol. To localize the expression of different cell types in the bat airway, IHC staining of bat trachea and bat lung were performed. Prior to the staining step, sample sections were deparaffinized and rehydrated. Subsequently, using 0.5% H_2_O_2_ diluted in ethanol to block endogenous peroxidase activity and 20% normal goat serum to block the nonspecific binding for 20 min at room temperature. Antibodies specific for acetylated tubulin (1:400, Sigma), mucin 5AC (1:200, Abcam, Cambridge, United Kingdom), and cytokeratin 5 (1:50, Abcam) were diluted in 1% bovine serum albumin (BSA). In addition, lectin staining using biotinylated Sambucus nigra (1:100, Vector Laboratories) and biotinylated Maackia amurensis (1:100, Vector Laboratories) was performed. The lectin staining method has been successfully applied in a number of previous publications by this group to detect sialic acids in porcine cells and tissues, the target cells of the viruses used in this study (37, 38, 63–65). Fixed samples were incubated with primary antibodies or lectin staining at 4°C overnight following a heat-induced antigen retrieval step, except for acetylated tubulin-specific IHC. After washing, the samples were incubated with biotinylated goat-anti-mouse IgG for acetylated tubulin- and mucin 5AC-specific IHC or goat-anti-rabbit IgG for cytokeratin 5-specific IHC diluted 1:200 (Vector Laboratories) as secondary antibodies for 45 min at room temperature. Next, the tissue sections were incubated with the peroxidase-conjugated avidin-biotin-complex (ABC method, PK-6100, VECTASTAIN Elite ABC kit; Vector Laboratories) for 20 min at room temperature. Finally, positive antigen-antibody reactions were visualized using 3,3′-diaminobenzidine tetrahydrochloride (DAB, Sigma-Aldrich, MO, USA) and H_2_O_2_, followed by counterstained with Mayer’s hematoxylin (Merck). All antibodies and lectins used in this study are listed in Table 1.

**TABLE 1:** Antibodies and lectins used in this study^*a*^

Antibody or stain	Source	Identifier or catalog no.	Species reactivity or sugar specificity
Antibodies			
Acetylated tubulin	Abcam	ab15568	Mouse, rat, human
Mucin5AC	Abcam	ab3649	Mouse, rat, human
Cytokeratin 5	Abcam	ab52635	Mouse, rat
Uteroglobin	Abcam	ab213203	Dog, human, mouse, rhesus, monkey
β-Catenin antibody	Abcam	ab264261	Mouse, human
ZO-1	Invitrogen	33-9100	Bovine, chicken, dog, fish, guinea pig, hamster, human, mouse, non-human primate, pig, rabbit, rat, sheep, tag, virus, *Xenopus*, zebrafish
IAV virus NP	Bio-Rad	MCA400	Virus
Alexa Fluor 488 goat anti mouse	Thermo Fisher Scientific	A-11001	Mouse
Alexa Fluor 568 goat anti-mouse	Thermo Fisher Scientific	A-11004	Mouse
Goat IgG anti-rabbit IgG (H + L)-Alexa Fluor 488	Biozol	111-545-045	Rabbit
Goat IgG anti-rabbit IgG (H + L)-Cy3	Biozol	111-165-003	Rabbit
Lectins			
MAA II	Vector Laboratories	B-1265-1	Alpha 2,3-linked sialic acid
SNA	Vector Laboratories	B-1305-2	Alpha 2,6-linked sialic acid

a IAV, influenza A virus; NP, nucleoprotein; MAA II, Maackia amurensis agglutinin II; SNA, Sambucus nigra agglutinin.

### Immunofluorescence analysis.

To characterize the cell types in bat AOs and bat airway ALI cell cultures, we performed immunofluorescence analyses. For bat AO immunofluorescence analysis, a 20-μL Matrigel drop containing bat AOs was seeded on a glass slide and was grown in 400 μL growth medium. At 4 days prior to immunofluorescence analysis, growth medium was changed to differentiation medium. The differentiated bat AOs on the glass slide and well-differentiated bat airway ALI were fixed with 3% paraformaldehyde for 30 min, permeabilized, and blocked with incubation medium containing 1% BSA, 5% goat serum, and 0.2% Triton X-100 at 37°C for 30 min. Next, the treated bat AOs and bat ALI were incubated with primary antibodies directed toward acetylated tubulin (1:100, Abcam), mucin 5AC (1:100, Abcam), cytokeratin 5 (1:100, Abcam), and uteroglobin (1:100, Abcam) with shaking at 4°C overnight. To characterize the integrity of differentiated samples on Transwell filters, the bat ALI samples were cultivated with anti-ZO-1 antibody (Life Technologies, Carlsbad, CA, USA) for staining tight junctions overnight. On the second day, the primary treated samples were washed three times with PBS, and secondary antibodies conjugated with green or red fluorescent stains (Alexa Fluor 488 and 568) were added (1:1,000, Life Technologies) in incubation medium for 1 h at room temperature. The nuclei were counterstained with DAPI (4′,6-diamidino-2-phenylindole).

To analyze the status of the swIAV infection, the AO and ALI samples were stained with an antibody directed against the influenza A virus NP (Bio-Rad, Oxford, United Kingdom). Tight junctions were visualized with an anti-β-catenin antibody (Sigma-Aldrich, St. Louis, MO, USA).

After mounting in ProLong Gold Antifade Mountant (Life Technologies), samples were analyzed using a Nikon Eclipse Ti-S microscope (Nikon, Tokyo, Japan) and a TCS SP5 confocal laser scanning microscope (Leica, Wetzlar, Germany). NIS-Elements Viewer 4.20 software (Nikon), LAS AF Lite software (Leica), and ImageJ/Fiji software were used to analyze images.

### Lectin staining.

The expression of sialic acids on bat airway tissue was analyzed by lectin staining. Biotinylated MAA II (Vector Laboratories) was used to detect alpha 2,3-linked sialic acid and SNA was used to detect alpha 2,6-linked sialic acid. The biotinylated lectin was visualized by fluorescence microscopy after incubation with Streptavidin-DyLight 488 for 1 h at room temperature (1:500, Vector Laboratories).

### Statistical analyses.

All *in vitro* experiments were conducted at least three times and data were subjected to statistical analysis using GraphPad Prism 8 software with Tukey’s multiple-comparison test. Results are shown as means with standard deviations. *P* < 0.05 was considered significant.
